# Optimizing Automated Optical Inspection: An Adaptive Fusion and Semi-Supervised Self-Learning Approach for Elevated Accuracy and Efficiency in Scenarios with Scarce Labeled Data

**DOI:** 10.3390/s24175737

**Published:** 2024-09-04

**Authors:** Yu-Shu Ni, Wei-Lun Chen, Yi Liu, Ming-Hsuan Wu, Jiun-In Guo

**Affiliations:** 1 Department of Electronics Engineering, Institute of Electronics, National Yang Ming Chiao Tung University, Hsinchu City 300, Taiwan; a321andy6314.ee12@nycu.edu.tw (Y.-S.N.); wlc310581001.10@nycu.edu.tw (W.-L.C.); 2 Software & Service Business Development Program Center, AUO Company, Taichung 407, Taiwan; yi.yl.liu@auo.com (Y.L.); minghsuan.mh.wu@auo.com (M.-H.W.)

**Keywords:** automatic optical inspection, object detection, semi-supervised learning

## Abstract

In the field of automatic optical inspection (AOI), this study presents innovative strategies to enhance object detection accuracy while minimizing dependence on large annotated datasets. We initially developed a defect detection model using a dataset of 3579 images across 32 categories, created in collaboration with a major Taiwanese panel manufacturer. This model was evaluated using 12,000 ambiguously labeled images, with improvements achieved through data augmentation and annotation refinement. To address the challenges of limited labeled data, we proposed the Adaptive Fused Semi-Supervised Self-Learning (AFSL) method. This approach, designed for anchor-based object detection models, leverages a small set of labeled data alongside a larger pool of unlabeled data to enable continuous model optimization. Key components of AFSL include the Bounding Box Assigner, Adaptive Training Scheduler, and Data Allocator, which together facilitate dynamic threshold adjustments and balanced training, significantly enhancing the model’s performance on AOI datasets. The AFSL method improved the mean average precision (mAP) from 43.5% to 57.1% on the COCO dataset and by 2.6% on the AOI dataset, demonstrating its effectiveness in achieving high levels of precision and efficiency in AOI with minimal labeled data.

## 1. Introduction

**Figure 1:**
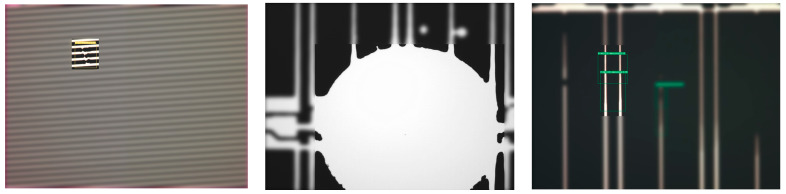
Detection result of different defects in AOI.

AOI has become an indispensable technology in the manufacturing sector, ensuring product quality and consistency by automatically detecting defects and anomalies across a wide range of products. As the manufacturing industry continues to advance, the demand for higher efficiency and precision in quality control processes has intensified. Consequently, AOI systems have become crucial for reducing reliance on labor-intensive manual inspections, thus achieving consistent product quality. Figure 1 illustrates typical defect detection results in AOI systems, including common defect types such as surface scratches, discolorations, and structural anomalies. These examples underscore the critical role of AOI in maintaining high production standards by effectively identifying and categorizing a diverse range of defects [1].

Despite these advancements, the development of high-accuracy object detection models in AOI systems still faces significant challenges, particularly the reliance on large, annotated datasets [2,3]. These datasets are essential for training models to accurately identify defects; however, the process of acquiring such extensive labeled data is often time-consuming and costly. This challenge is further compounded in scenarios in which labeling requires expert knowledge or in which defects are subtle and diverse, leading to additional complications in model training. This brings us to a crucial point: the need for innovative methods that can overcome these limitations and enhance the efficiency of AOI systems [4,5,6].

To address these significant hurdles, this study introduces two innovative strategies aimed at enhancing object detection model accuracy while minimizing dependence on extensive annotated datasets. The proposed approach leverages the concepts of semi-supervised learning and adaptive learning, enabling the more efficient use of available data and facilitating self-learning within models [7,8]. These methodologies not only address the limitations of traditional object detection models but also contribute to the broader field of machine learning by demonstrating how self-learning and adaptive strategies can be applied in industrial applications [9].

The AFSL method is a central innovation in this study, designed to optimize AOI systems by improving their ability to learn from both labeled and unlabeled data. This method is particularly relevant given the previously mentioned challenges of limited labeled data and the complexity of defect detection [10,11,12]. AFSL is structured around several key components, each with a specific purpose:

- Bounding Box Assigner: This component is responsible for generating and refining the bounding boxes used to identify defects within images. It uses an intersection over union (IOU) filter to ensure that only the most reliable bounding boxes are passed on for further training [13]. This step is critical for reducing errors in the early stages of the model’s learning process [14];
- Adaptive Training Scheduler: To further refine the training process, this scheduler dynamically adjusts the learning rate and other parameters based on the confidence levels of the pseudo-labels generated during training. By doing so, it helps to prevent the model from being misled by unreliable data, which is a common challenge in semi-supervised learning [15,16,17];
- Data Allocator: This module applies various data augmentation techniques to both labeled and unlabeled datasets, increasing the diversity of the training data. The goal is to expose the model to a wide range of possible defect scenarios, thereby improving its ability to generalize across different types of products and manufacturing conditions [18,19].

While AFSL offers several innovative solutions, it is important to acknowledge the challenges associated with its implementation. One of the primary challenges involves managing the quality of pseudo-labels generated during the semi-supervised learning process. Inaccurate [20,21] or noisy pseudo-labels [22,23] can mislead the model, potentially degrading its performance. Additionally, balancing the training process to ensure that the model effectively utilizes both labeled and unlabeled data without overfitting to the more confident labels is another significant hurdle. These challenges highlight the importance of the careful implementation and ongoing refinement of the AFSL methodology to achieve optimal results.

In the initial phase of this research, a preliminary defect detection model was developed using a dataset of 3579 images across 32 categories, provided by a leading panel manufacturer in Taiwan. The model’s performance was subsequently evaluated on a set of 12,000 ambiguously labeled images, which presented a significant challenge due to the variability and complexity of the defects. Through iterative refinement processes, including data augmentation and annotation correction, the model’s accuracy and generalizability were substantially improved.

Building on these foundational improvements, the second phase introduced the AFSL method to further enhance the self-learning capabilities of the AOI system. While a detailed evaluation of the results, such as the improvements in the model’s mean average precision (mAP), will be discussed in the results section, it is noteworthy that AFSL played a critical role in achieving significant performance gains. Specifically, the mAP improved from 43.5% to 57.1% on the COCO dataset and by 2.6% on an in-house AOI dataset. Furthermore, recent studies have shown that incorporating reinforcement learning and transfer learning into the AFSL framework could further improve defect detection accuracy and adaptability across different manufacturing environments. These preliminary indicators suggest the potential of AFSL to revolutionize AOI systems, which will be explored in greater detail later in this thesis.

The primary objective of this research is to enhance the accuracy and efficiency of object detection models within AOI systems while reducing the reliance on large, annotated datasets. This goal is achieved through the development of the AFSL method, which integrates semi-supervised and adaptive learning techniques to optimize the use of both labeled and unlabeled data [24,25].

The main conclusions of this study highlight the significant improvements in model performance achieved through these methods. While detailed results will be provided in subsequent sections, it is evident that the AFSL approach not only enhances accuracy but also offers a scalable framework for future AOI systems. By addressing the critical challenge of limited labeled data, this research paves the way for more efficient, cost-effective, and scalable AOI solutions in the manufacturing industry.

## 2. Related Work

The field of AOI has experienced tremendous advancements over the past few decades, driven by the increasing demand for efficient and accurate quality control mechanisms in manufacturing. Various methodologies have been explored to enhance the performance of AOI systems, particularly in the detection and classification of defects. This section provides an in-depth review of the key contributions and methodologies that have shaped the current landscape of AOI, highlighting their strengths and limitations.

### 2.1. Traditional Machine Learning Approaches

Traditional machine learning techniques have laid the groundwork for defect detection in AOI systems. Techniques such as support vector machines (SVMs), k-nearest neighbors (k-NNs), and decision trees have been extensively utilized for defect classification based on extracted features from images. Kang et al. (2009) employed SVMs to detect defects in thin-film-transistor liquid-crystal display (TFT-LCD) panels, optimizing feature selection and classification parameters to achieve a notable accuracy [26]. Similarly, Iwahori (2017) utilized a k-NN algorithm for defect detection in electronic circuit boards, in which keypoint extraction and CNN features played a critical role in identifying defect patterns [27]. Additionally, Akbar et al. (2013) used decision trees for detecting surface defects, demonstrating the efficacy of rule-based classification methods [28]. Traditional machine learning approaches also explored various feature extraction techniques, such as wavelet transforms and Gabor filters, which were crucial in the early stages of AOI development [29]. However, these methods often required manual feature extraction, which was labor-intensive and limited in capturing the full complexity of defect patterns. This constraint posed challenges in the scalability and adaptability of these models in dynamic manufacturing environments, which this research addresses by automating feature extraction through deep learning techniques. Future trends in this area may involve the integration of traditional machine learning with newer technologies, such as edge computing, to enhance real-time processing capabilities in AOI systems.

### 2.2. Deep-Learning-Based Methods

The advent of deep learning has brought a paradigm shift in AOI systems, enabling more automated and accurate defect detection. CNNs, in particular, have become the cornerstone of modern AOI systems due to their ability to learn hierarchical feature representations directly from raw image data. Ren et al. (2018) proposed a deep-learning-based approach for automated surface inspection, significantly outperforming traditional methods in terms of accuracy and robustness [30]. Similarly, Yuan-Fu (2019) developed a deep learning model for identifying defect patterns in semiconductor wafers, achieving remarkable improvements over conventional machine learning models [31]. Beyond CNNs, other deep learning architectures like recurrent neural networks (RNNs) and generative adversarial networks (GANs) have been explored for AOI applications. For instance, Zheng et al. (2020) applied a semi-supervised deep learning approach for automated surface inspection, addressing the challenge of data scarcity in AOI systems [32]. GANs have also been used to generate synthetic defect data for training purposes, further tackling the issue of limited labeled datasets [33].

Despite their success, deep learning models require large amounts of annotated data for training, posing a significant challenge in scenarios in which acquiring labeled datasets is expensive and time-consuming. This study builds upon these foundations by introducing semi-supervised and adaptive learning methods to reduce dependency on large labeled datasets, making deep learning more accessible and efficient for AOI applications. Looking ahead, the combination of deep learning with transfer learning and meta-learning could further reduce the need for extensive labeled datasets, accelerating the deployment of AOI systems across various manufacturing environments.

### 2.3. Semi-Supervised Learning

SSL has emerged as a promising approach to address the limitations of traditional supervised learning by leveraging both labeled and unlabeled data [34]. SSL techniques aim to improve model performance using a limited amount of labeled data, augmented by a larger pool of unlabeled data. Richter et al. (2017) provided a comprehensive review of SSL methods and their applications, demonstrating the effectiveness of SSL in various domains, including AOI [35]. Their study highlighted how SSL can reduce the dependency on labeled data while maintaining a high accuracy. Hung et al. (2022) introduced a semi-supervised learning approach for defect detection in PCBs, showcasing how SSL can be effectively applied to scenarios in which labeled data are scarce and expensive to obtain [36]. Ebayyeh et al. (2020) further explored SSL in the context of PCB quality inspection, integrating a data-expanding strategy to improve defect detection accuracy [37]. The use of pseudo-labeling in SSL, in which the model generates labels for unlabeled data, has also been explored to boost the training process in AOI systems. This method has been shown to significantly enhance defect detection performance in manufacturing lines with limited labeled data [38]. However, SSL is not without its challenges; for instance, model degradation is possible if the pseudo-labels generated are inaccurate. This study addresses these limitations by integrating adaptive learning techniques that dynamically adjust the training process based on the confidence of the pseudo-labels, thereby improving the reliability and robustness of SSL in AOI. Future research could explore the integration of SSL with active learning, in which the model selectively queries the most informative data points for labeling, further enhancing the efficiency of AOI systems.

### 2.4. Adaptive Learning and Self-Learning Techniques

Adaptive learning and self-learning techniques represent significant advancements in AOI systems, enabling models to continuously improve and adapt to new data. Adaptive learning involves dynamically adjusting model parameters and learning rates based on the characteristics of the data, while self-learning allows models to iteratively refine their predictions using feedback from unlabeled data. Sun et al. (2023) proposed a continual learning framework that adapts defect classification and inspection processes dynamically, significantly improving the model’s performance by focusing on varying defect types [39]. This approach balances the trade-off between precision and recall, making it highly effective in environments in which defect types are diverse and challenging.

Self-learning techniques, such as the use of reinforcement learning for AOI, have also gained attention. Landgraf et al. (2021) applied reinforcement learning to optimize the inspection paths of AOI systems, resulting in faster and more accurate defect detection [40]. Similarly, Jing et al. (2018) developed a computational framework that combines coverage planning with reinforcement learning for automatic path generation in robotic inspection tasks, further enhancing the efficiency and accuracy of AOI systems [41]. These techniques have demonstrated their effectiveness in improving the robustness and generalizability of AOI systems, particularly in environments with limited labeled data. However, adaptive and self-learning methods can be complex to implement and may require significant computational resources.

This research contributes by optimizing these techniques for use in AOI, making them more practical and scalable in industrial applications. In the future, advancements in hardware, such as the use of specialized AI chips, could further enhance the efficiency of adaptive and self-learning methods in AOI, enabling real-time adaptation and decision making.

### 2.5. Hybrid Approaches

Recent research has also explored hybrid approaches that combine multiple methodologies to leverage their respective strengths. Schlosser et al. (2022) developed a hybrid model that integrates deep learning with traditional machine learning techniques for defect detection in semiconductor manufacturing. This approach combined the feature extraction capabilities of a CNN with the classification accuracy of traditional machine learning methods, resulting in a highly effective defect detection system [42]. Similarly, Chu et al. (2022) proposed a hybrid-learning framework that utilizes both deep learning and edge computing for operational visual quality inspection within Internet of Things (IoT) systems. Their method effectively combines the strengths of different approaches to enable real-time defect detection in manufacturing environments [43].

These hybrid models offer the potential to overcome the limitations of single-method approaches, providing more versatile and powerful solutions for AOI. Despite their benefits, hybrid models can be challenging to design and require the careful balancing of the different techniques involved. This study advances the field by proposing a hybrid approach that effectively integrates semi-supervised learning with adaptive learning, thereby overcoming the limitations of existing models and improving defect detection accuracy in AOI systems. Looking forward, the combination of hybrid models with cloud computing and the IoT could revolutionize AOI by enabling more flexible, scalable, and connected inspection systems [44].

### 2.6. Challenges and Future Directions

While significant progress has been made in AOI systems, several challenges remain. One of the primary challenges is the scarcity of labeled data, which limits the training of deep learning models. Additionally, the variability and complexity of defect patterns pose significant challenges for model generalization. This research addresses these challenges by leveraging semi-supervised and adaptive learning techniques that minimize dependency on labeled data while enhancing model generalization. Moreover, the integration of domain knowledge and expert insights into AI models can further enhance their performance and reliability. Leveraging advancements in explainable AI (XAI) could also provide valuable insights into the decision-making processes of AOI systems, enabling better interpretability and more trust in automated inspections. In summary, the advancements in machine learning, particularly deep learning, semi-supervised learning, and adaptive learning, have significantly enhanced the capabilities of AOI systems. However, ongoing research and innovation are essential to overcome the remaining challenges and unlock the full potential of AOI technology in industrial applications. By continuously refining these methodologies and exploring new hybrid approaches, we can pave the way for more accurate, efficient, and scalable AOI systems in the future.

## 3. Research Methodology

**Figure 2:**
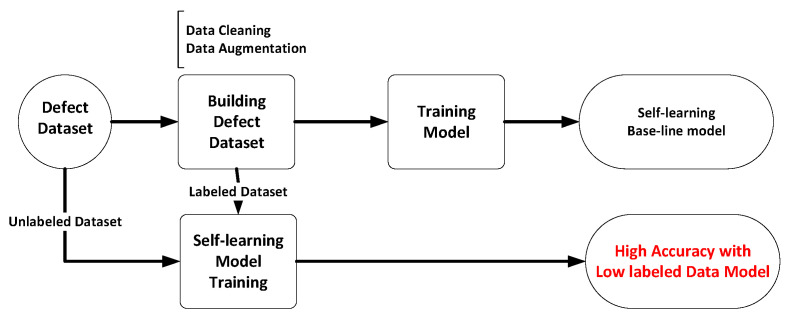
The proposed system workflow.

In this study, we focused on developing a robust AOI system starting from the initial stages of the manufacturing process. The proposed approach involved multiple phases, including initial dataset assembly, model development, data augmentation, semi-supervised learning implementation, and thorough evaluation. This comprehensive design aimed to enhance the accuracy of defect detection while minimizing reliance on extensive annotated datasets. The complete process of the proposed method is illustrated in Figure 2, showcasing everything from early data establishment to subsequent model development, which involves creating a foundational dataset and model, validating labeled images, and then integrating both labeled and unlabeled images to establish a self-learning model. This allows us to achieve better results using a minimal amount of labeled data.

### 3.1. Initial Dataset Assembly

The initial dataset was provided by our industrial collaborators and consisted of 3579 images encompassing 32 defect categories. These images, captured by high-resolution AOI systems, included various defect types such as scratches, discolorations, and structural anomalies. Each image was meticulously annotated by expert inspectors to ensure high-quality labels. This dataset served as the foundation for our initial defect detection model.

The collected data have been modified in order to avoid exposing company secrets. However, to ensure the repair status is clearly identified, the defect area of defective image has been left unmodified.

The first version of our defect detection model was constructed using the initial dataset. We employed a YOLOv7 [45] base model architecture tailored for object detection tasks. The key components of the model included the following:

- Input Layer: High-resolution images (input size 512 × 512 pixels);
- Feature Extraction Layers: Multiple convolutional layers with ReLU activation functions and max-pooling layers;
- Region Proposal Network (RPN): Generates region proposals where defects are likely to be located;
- Classification and Regression Heads: Predicts defect categories and refines bounding box coordinates.

The model was trained using the initial dataset with a stochastic gradient descent (SGD) optimizer at a learning rate of 0.001. The loss function combined classification loss (cross-entropy) and localization loss (smooth L1 loss). Training was conducted over 50 epochs with a batch size of 16.

Following the initial training, the model was evaluated using a set of 12,000 ambiguously labeled images. This evaluation aimed to assess the model’s baseline performance and verify the accuracy of the initial annotations. Key performance metrics such as precision, recall, F1-score, and mAP were computed to gauge the model’s effectiveness.

### 3.2. Dataset Adjustment

**Figure 3:**
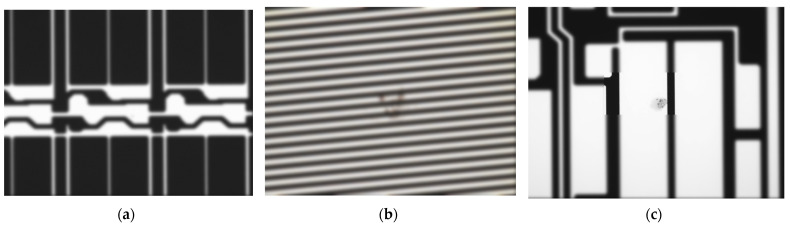
The differences between the (**a**) in-plane region, (**b**) the GOA region, and (**c**) the Fan-out region.

After refining the initial dataset and model, we identified some minor errors. Although in-plane and out-of-plane defects were categorized together, their characteristics were significantly different, as shown in Figure 3. We separated the original in-plane defects from the GOA region and the Fan-out region, and finally used post-processing to reclassify the categories. This method helps to improve the overall accuracy of the classification.

### 3.3. Data Augmentation and Annotation Refinement

**Figure 4:**
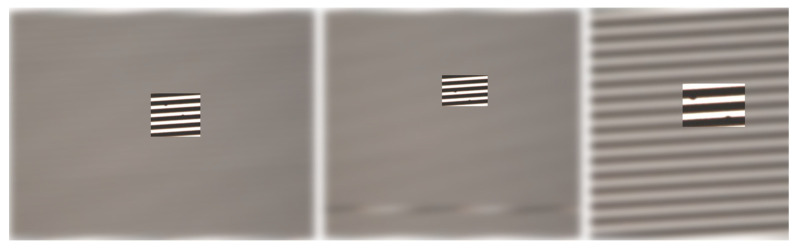
Random scaling of the image.

To enhance the dataset’s diversity and improve the model’s generalizability, various data augmentation techniques were employed. These included the following:

- Scaling: Random scaling within a range of 0.8 to 1.2 times the original size as shown in Figure 4;
- Flipping: Horizontal and vertical flips;
- Brightness and Contrast Adjustment: Random changes in brightness and contrast levels;
- Color Adjustment: Applying different color mappings to simulate various imaging conditions.

In addition to data augmentation, the ambiguously labeled dataset underwent an iterative annotation refinement process. Expert inspectors reviewed and corrected the annotations based on the model’s predictions, ensuring high-quality labels. This refinement included re-annotating bounding boxes and adjusting annotation sizes to improve precision.

### 3.4. Implementation of Adaptive Fusion Semi-Supervised Self-Learning

To address the limitations of labeled data, we implemented the AFSL method. AFSL is designed to enhance the model’s ability to learn from both labeled and unlabeled data, facilitating continuous improvement and reducing human annotation efforts. The following sections will discuss the key components of AFSL.

### 3.5. Semi-Supervised Learning Object Detection

**Figure 5:**
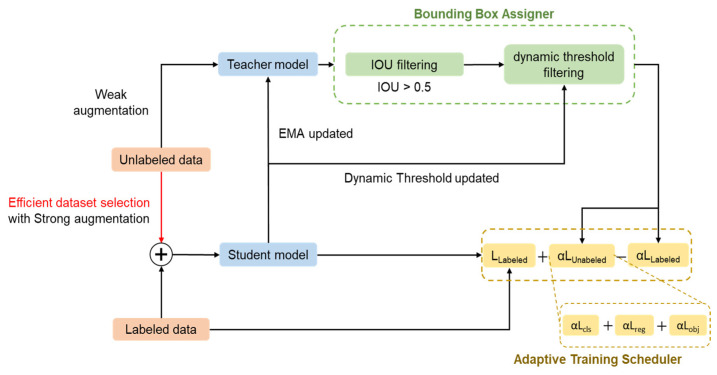
Proposed AFSL system architecture diagram.

Firstly, AFSL will be introduced in detail. Figure 5 shows the architecture of AFSL. The labeled dataset also participates in AFSL training to guide model training. Our proposal integrates three blocks with several innovations into AFSL based on the Mean Teacher [46] teacher–student architecture. These are the Bounding Box Assigner, Adaptive Training Scheduler, and various training strategies. First, a robust pseudo-label needs to be generated, so the robustness of the complex model is crucial. AFSL fine-tunes the complex model to generate robust pseudo-labels. Once the complex model is sufficiently robust, self-learning training fine-tunes the target model. The ΔmAP is used to judge the AFSL complex model’s fine-tuning. If ΔmAP is larger than 1% in the first three epochs, it indicates the complex model needs improvement for the newly added dataset, and AFSL continues training. Conversely, if ΔmAP is less than 1% in the first three epochs, it means the complex model is robust enough, and AFSL will stop, starting the one-shot self-learning algorithm.

The following sub-sections will dive into the details of each AFSL method.

#### 3.5.1. Complex Model Selection

Before diving into the architecture of AFSL, the backbone of AFSL will be introduced. To generate robust pseudo-labels, a powerful model is as crucial as an excellent semi-supervised learning algorithm. This paper focuses on the object detection anchor-based model due to its efficiency and robustness. YOLOv7 [45], one of the powerful and lightweight representative object detection anchor-based models, is known for its performance on open dataset validation and its integrated training methodology with previous augmentation and optimizer strategies.

Table 1 shows the performance and complexity comparison of YOLOv7 with other object detection models. YOLOv7 has higher AP50:95 and less model complexity. Additionally, YOLOv7 has an improved performance due to applying several training strategies, such as Mosaic, which improve AP@50 by 1.8%.

#### 3.5.2. Bounding Box Assigner

Similar to traditional supervised learning, semi-supervised learning for the student model contains pseudo-labels. An adaptive unlabeled loss is proposed, replacing fixed coefficients with bounding box confidence. We proposed a dynamic and adaptive unlabeled loss function, improving the training process and balancing student model learning from labeled and unlabeled data. The proposed method prevents student models from learning unreliable pseudo-labels, ensuring effective training.

The Adaptive Training Scheduler includes both labeled loss and unlabeled loss. It optimizes the unlabeled loss function for semi-supervised object detection by using confidence weighting, allowing the model to assess the quality of pseudo-labels. This approach improves the model’s efficiency and accuracy during training. Additionally, a debiasing penalty is incorporated to reduce uncertainties during training, balancing the influence of labeled and unlabeled data.

#### 3.5.3. Data Allocator and Adaptive Training Scheduler

In semi-supervised object detection, implementing an effective training strategy that works in tandem with the Bounding Box Assigner and Adaptive Training Scheduler is essential for maintaining training stability. This thesis introduces both strong and weak augmentation techniques for a consistency-based approach on the unlabeled dataset, aiding the inference processes of the teacher and student models.

**Figure 6:**
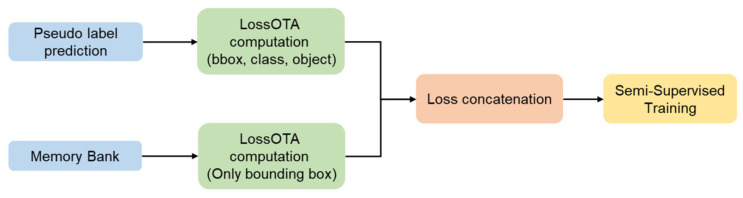
The block diagram of mean bounding box loss.

**Figure 7:**
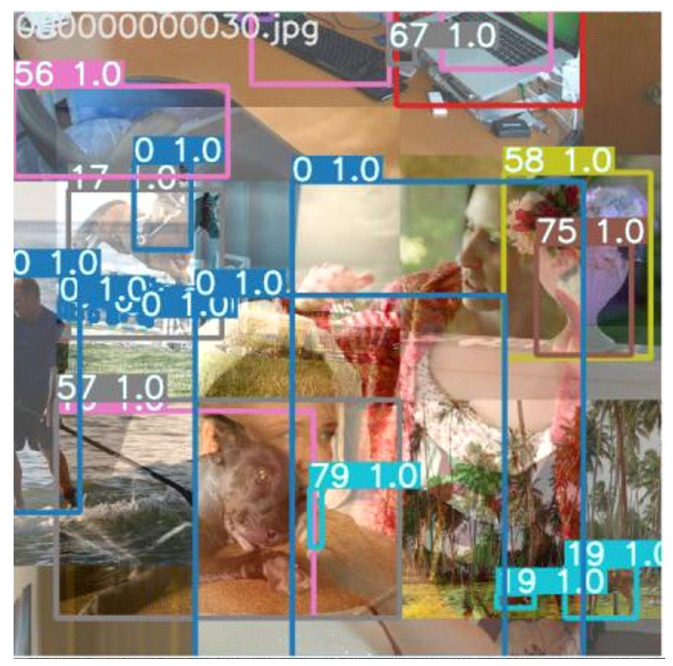
The training image for student after YOLOv7 Mosaic was implemented.

Figure 6 depicts the block diagram of the Adaptive Fusion Semi-Supervised Learning system, highlighting the use of weak and strong augmentations for input data. Weak augmentation techniques, such as horizontal flips and Gaussian noise, are used to modify the unlabeled data without altering essential image features. For strong augmentation, methods like Mosaic with HSV adjustments, Mixup, and Cutout—techniques utilized in YOLOv7—are employed to enhance the student model’s training. Figure 7 provides an example of a training image after applying Mosaic augmentation.

Additionally, we proposed using the Efficient Teacher method to ensure smoother fitting of the student model during training [47]. Early in the training process, high learning rates and improperly fitted unlabeled data can result in empty labels for challenging frames. To address this, we isolate these empty-labeled frames from the training process of the current epoch and reintegrate them once they are properly labeled.

## 4. Performance Evaluation and Validation

In this section, we present a comprehensive evaluation of the performance and validation of our object detection models. We utilized various techniques to enhance the dataset’s diversity and improve the model’s generalizability. The proposed approach includes detailed data augmentation and annotation refinement, which are crucial steps to ensure the robustness and accuracy of the models. The effectiveness of these techniques is demonstrated through a performance comparison of different editions of our models, including the YOLOv7 model.

### 4.1. Data Augmentation and Annotation Refinement

To improve the model’s performance, various data augmentation techniques were employed, and the ambiguously labeled dataset underwent iterative annotation refinement. These steps were crucial in enhancing the model’s accuracy and generalizability as shown in Table 2 and Table A1 included in Appendix A.

### 4.2. AFSL Validation on COCO and AOI Datasets

The following results validate the proposed AFSL method, starting with the COCO dataset and followed by the AOI dataset.

#### 4.2.1. COCO Dataset Validation

In this section, the COCO Standard dataset serves as a benchmark for validating the AFSL methodology. Before we dive into the experimental results, Equation (1) introduces an evaluation matrix designed to normalize the results from other research studies for comparison. This equation is particularly important in scenarios like AOI, in which both model performance and computational complexity play a crucial role, especially given the constraints of edge computing.

The relationship between model performance and complexity is critical in AOI systems because these systems often operate in environments in which real-time processing is required, and computational resources are limited. By normalizing the complexity of the model (measured in FLOPs) and the labeled data ratio, Equation (1) allows us to compare the efficiency and effectiveness of our proposed AFSL method against other approaches. This comparison is especially relevant for semi-supervised object detection tasks, for which reducing the amount of labeled data while maintaining or improving performance is a key objective.

Equation (1) is formulated as follows:

(1) $$EMAFSL=\frac{{map}_{@0.5-0.95}Improment(\%)\times 100}{Labeled\;Ratio(\%)\times Model\;Complexity(G\;FLOPs)}$$

This equation helps demonstrate that a model with lower FLOPs that consumes fewer computational resources and requires a shorter training period can achieve a superior performance in semi-supervised object detection. The normalization of the labeled ratio is optional when the labeled data ratio is the same across different models; in such cases, we only normalize the model’s complexity to focus on the computational efficiency.

The inclusion of model complexity, specifically measured in FLOPs, is justified by the practical demands of AOI systems, which often rely on edge computing. In these scenarios, it is essential that the models are not only accurate but also computationally efficient. Edge devices typically have limited processing power, and a model with a lower computational footprint (i.e., lower FLOPs) is more suitable for real-time applications. Thus, Equation (1) provides a comprehensive evaluation by considering both the accuracy and the computational demands of the model, ensuring that the proposed AFSL method is both effective and efficient in real-world AOI applications.

To further validate the scientific correctness of Equation (1), we will conduct additional experiments comparing the results of different models on the COCO dataset using this evaluation metric, as shown in Table 3. These experiments will help confirm that the equation appropriately reflects the trade-offs between accuracy and computational efficiency, particularly in the context of edge computing in AOI systems.

As shown in Table 3, we observe that the performance of our proposed AFSL not only excels in the one-stage anchor-based method but also achieves the highest *EMAFSL* value across all SSOD methodologies when complexity is considered. The method with the highest score other than AFSL in the 10% labeled data category is Dense Teacher [48], which achieved a score of 7.01. However, our proposed AFSL method had a score of 8.63, which is 1.62 points higher than that of Dense Teacher [48]. This indicates that the AFSL methodology we proposed is highly competitive in terms of performance and training time consumption.

**Table 3:** The performance for CNN-based semi-supervised learning on object detection under 10% labeled COCO Standard dataset (* means that the method is conducted on the backbone of Efficient Teacher [47]).

Methodology		FLOPs	AP@50:95 (%)	EMAFSL
Two-stage anchor-based	Baseline	202.31G	23.6	
	STAC [24]		28.64	2.49
	Instant Teaching [49]		30.40	3.36
	Humble Teacher [50]		31.61	3.96
	Unbiased Teacher [51]		31.50	3.90
	Soft Teacher [52]		34.04	5.16
	LabelMatch [53]		35.49	5.87
	PseCo [54]		36.06	6.15
One-stage anchor-free	Baseline	200.59G	23.07	
	Unbiased Teacher v2 [55]		32.61	4.76
	DSL [56]		36.22	6.56
	Dense Teacher [48]		37.13	7.01
One-stage anchor-free	Baseline	169.61G	24.04	-
	Unbiased Teacher * [57]		30.34	3.71
	Efficient Teacher [47]		34.09	5.92
	Baseline	**104.7G**	25.16	-
	AFSL (ours)		34.20	**8.63**
	Fully Supervised		49.7	-

As detailed in Table 4, the AFSL training improved the mAP@0.5 from 43.5% to 57.1% when using 10% labeled data, compared to the baseline model. Additionally, we incorporated the COCO Additional dataset to explore whether supplementing with more data could further enhance the model’s performance. The results indicated that adding the COCO Additional dataset improved the mAP@0.5:0.95 by 1.5%, compared to the fully supervised model trained solely on the COCO Standard dataset, demonstrating AFSL’s significant potential for continuous learning.

#### 4.2.2. AOI Dataset Validation

Furthermore, we conducted experiments on the AOI dataset provided by AUO, as shown in Table 5. We experimented with 10% and 25% labeled datasets. The results demonstrate that even with just 10% labeled data, AFSL exhibits a high level of adaptability and continuous improvements. When the labeled dataset proportion was increased to 25%, the AFSL-trained model outperformed the fully supervised results, further proving AFSL’s powerful adaptive training capabilities.

Table 6 and Table A2 show the data for various categories in the AOI dataset based on 25% labeled data after AFSL training. The black bars represent the fully supervised results, and the white bars represent the AFSL training results. The table indicates that AFSL not only improved the mAP@0.5:0.95 by 2.6%, but also maintained stable training across various categories.

Most categories performed slightly better in comparison to the fully supervised results, confirming that AFSL effectively captures the characteristics of each category. Additionally, AFSL can handle data imbalance to some extent, mitigating the issues faced by traditional semi-supervised object detection methods.

## 5. Conclusions

The proposed method significantly enhanced the accuracy of AOI systems while reducing reliance on extensive annotated datasets. Beginning with an initial dataset of 3579 images spanning 32 defect categories, we developed and refined a defect detection model, which was subsequently validated on 12,000 ambiguously labeled images. Through iterative processes of data augmentation and annotation refinement, the model’s accuracy saw a substantial improvement.

Our experiments demonstrated that the AFSL method achieved remarkable enhancements. On the COCO dataset, the AFSL method increased mAP@0.5:0.95 from 43.5% to 51.7% using only 10% labeled data. Similarly, on the AOI dataset, the AFSL method with 25% labeled data outperformed the fully supervised results, showing a 2.6% improvement in mAP@0.5:0.95. Despite these advancements, challenges such as the scarcity of labeled data and the complexity of defect patterns persist. Future research should focus on developing more effective data augmentation techniques and integrating domain knowledge. In the AOI field, particularly in defect detection, relying solely on image data often proves insufficient due to the heavy reliance on expert experience. Thus, combining multi-modal models with self-learning techniques could potentially yield even more significant results.

In conclusion, this paper proposes a scalable and efficient approach for AOI systems, markedly improving defect detection accuracy and efficiency. The AFSL method provides a robust solution for reducing annotation efforts while achieving a high precision, laying a strong foundation for future advancements in manufacturing quality control and automation.

## Figures and Tables

**Table 1:** The performance and complexity comparison of YOLOv7 and other object detection models.

Method	Resolution	Mosaic	Param.	FLOPs	AP50:95 (%)	Method	Resolution	Mosaic	Param.
FRCNN	[1333,800]	X	39.8M	202.31G	40.3	FRCNN	[1333,800]	X	39.8M
FCOS	[1333,800]	X	32.02M	200.59G	38.5	FCOS	[1333,800]	X	32.02M
YOLOv5w/o	[640,640]	X	42.56M	109.59G	41.2	YOLOv5w/o	[640,640]	X	42.56M
YOLOv5	[640,640]	✓	42.56M	109.59G	49	YOLOv5	[640,640]	✓	42.56M
YOLOv7	[640,640]	✓	37.62M	106.59G	51.5	YOLOv7	[640,640]	✓	37.62M
RetinaNet	[1333,800]	X	37.74M	239.32G	39.5	RetinaNet	[1333,800]	X	37.74M
DenseNet	[640,640]	✓	42.13M	169.61G	44.86	DenseNet	[640,640]	✓	42.13M

**Table 2:** Performance comparison of YOLOv7 with different refinement stages.

Datasets	Model Accuracy
No modifications	84.00%
First Edition (Category Refinement)	83.50%
Second Edition (Category Refinement and Data Augmentation)	91.30%
Final Edition (Category Refinement, Data Augmentation, and Full Data Reorganization)	**93.88%**

**Table 4:** The performance and complexity comparison of YOLOv7 and other object detection models [51] using COCO dataset for AFSL validation with 10% labeled data.

	Precision	Recall	AP @0.5	AP @0.5:0.95
Yolov7_640 × 640 (Baseline 10% labeled)	35.40%	31.80%	43.50%	25%
Yolov7_640 × 640 (AFSL 10% labeled)	51.70%	45.30%	57.10%	38.10%
Yolov7_640 × 640 (AFSL 100% labeled, additional)	62.50%	58.70%	70.4%	50.5%
Yolov7_640 × 640 (Fully Supervised)	63.20%	56.20%	69.70%	49%

**Table 5:** AOI dataset for AFSL validation with different ratios of labeled data.

Labeled Ratio	Precision	Recall	AP @0.5	AP @0.5-0.95
Yolov7_640 × 640(Baseline 10% labeled)	70.70%	76.50%	78.80%	46.70%
Yolov7_640 × 640(Semi-supervised 10% labeled)	79.60%	83.70%	84.7% (+5.9%)	61.4% (+14.7%)
Yolov7_640 × 640(Semi-supervised 10% labeled)	81.40%	82.20%	86.70%	53.20%
Yolov7_640 × 640(Semi-supervised 25% labeled)	92.70%	95.40%	97% (+0.2%)	73% (+2.6%)
Yolov7_640 × 640 (Fully Supervised)	92.10%	94.60%	96.80%	70.40%

**Table 6:** AFSL training under 25% labeled AOI dataset: a comparison between supervised learning (Baseline) and AFSL.

Item	Precision	Precision	AP@ 0.5	AP@ 0.5	AP@ 0.5:0.95	AP@ 0.5:0.95)
Method	Baseline	AFSL	Baseline	AFSL	Baseline	AFSL
Accuracy	0.921	0.927	0.968	0.97	0.704	0.73

## Data Availability

Data are contained within the article.

## Appendix A

**Table A1:** Detailed performance comparison of YOLOv7 with different refinement stages.

Defect Name	Original	First Edition	Second Edition	Final Edition
Type1	79.70%	76.60%	84%	**87.1%**
Type2	87.50%	85.20%	100%	**100%**
Type3	57.20%	82.10%	88%	**97%**
Type4	97.80%	82.70%	93%	93%
Type5	95.30%	82.90%	86%	**100%**
Type6	89.60%	99.40%	100%	**98.1%**
Type7	100.00%	100.00%	100%	**100%**
Type8	97.70%	60.90%	74%	91.3%
Type9	89.20%	86.70%	100%	**100%**
Type10	89.60%	92.50%	93%	**93%**
Type11	85.40%	85.30%	94%	**94%**
Type12	83.80%	93.30%	100%	**100%**
Type13	83.70%	96.10%	100%	**100%**
Type14	77.40%	83.70%	100%	**100%**
Type15	94.50%	94.00%	98%	**98%**
Type16	92.70%	100.00%	100%	**100%**
Type17	81.10%	75.90%	93%	**93%**
Type18	88.20%	30.80%	56%	75%
Type19	78.70%	75.50%	77%	**84%**
Type20	74.20%	76.30%	76%	**76%**
Type21	74.40%	82.80%	86%	**86%**
Type22	75.80%	87.10%	93%	**93%**
Type23	68.80%	88.00%	82%	**82%**
Type24	72.70%	70.20%	84%	**84%**
Type25	86.70%	86.90%	97%	**97%**
Type26	88.50%	93.30%	94.6%	**94.6%**
Type27	67.30%	58.90%	82%	**87%**
Type28	87.30%	89.20%	95%	**95%**
Type29	84.70%	87.30%	98%	**98%**
Type30	85.60%	69.10%	83%	**92%**
Type31	100.00%	100.00%	100%	100%
Type32	71.70%	78.40%	92%	**93.5%**
Total	84.00%	83.50%	91.30%	**93.88%**

## Appendix B

**Table A2:** Detailed performance metrics of various defect types between baseline model and AFSL model.

Defect Name	Precision	Precision	AP (0.5)	AP (0.5)	AP (0.5-0.95)	AP (0.5-0.95)
	Baseline	AFSL	Baseline	AFSL	Baseline	AFSL
Total	0.921	0.927	0.968	0.97	0.704	0.73
Type1	0.843	0.869	0.966	0.966	0.588	0.633
Type2	0.879	0.91	0.986	0.993	0.672	0.711
Type3	0.967	0.95	0.993	0.992	0.992	0.987
Type4	0.972	1	0.992	0.995	0.711	0.746
Type5	0.978	0.988	0.992	0.993	0.988	0.988
Type6	0.977	0.978	0.995	0.995	0.695	0.732
Type7	0.992	0.992	0.995	0.995	0.802	0.842
Type8	0.999	0.998	0.995	0.995	0.995	0.994
Type9	0.767	0.823	0.966	0.97	0.737	0.775
Type10	0.929	0.934	0.976	0.981	0.584	0.607
Type11	0.943	0.947	0.963	0.971	0.656	0.712
Type12	0.788	0.819	0.89	0.912	0.567	0.594
Type13	1	0.997	0.995	0.95	0.832	0.847
Type14	0.95	967	0.973	0.982	0.521	0.554
Type15	O.927	0.936	0.99	0.993	0.75	0.773
Type16	0.805	0.788	0.751	0.748	0.523	0.521
Type17	0.737	0.769	0.826	0.859	0.486	0.524
Type18	0.937	0.936	0.978	0.981	0.803	0.822
Type19	0.903	0.915	0.976	0.974	0.58	0.616
Type20	0.934	0.964	0.978	0.982	0.813	0.837
Type21	0.859	0.862	0.973	0.976	0.641	0.66
Type22	1	1	0.995	0.995	0.837	0.859
Type23	0.994	0.993	0.995	0.995	0.752	0.762
Type24	0.902	0.905	0.978	0.985	0.682	0.728
Type25	0.881	0.89	0.979	0.939	0.612	0.63
Type26	0.957	0.942	0.991	0.992	0.827	0.846
Type27	0.95	0.959	0.985	0.994	0.655	0.696
Type28	0.997	0.3998	0.995	0.995	0.754	0.804
Type29	0.919	0.877	0.984	0.973	0.59	0.622
Type30	0.742	0.933	0.946	0.949	0.585	0.608
Type31	1	1	0.995	0.995	0.546	0.597
Type32	0.849	0.839	0.986	0.981	0.694	0.733
